# P15 Management of Takayasu arteritis during pregnancy: two district general hospital case studies highlighting tailored treatment plans

**DOI:** 10.1093/rap/rkae117.046

**Published:** 2024-11-05

**Authors:** Zainab Arif, Blossom Cooke, Alexandra Tillett, Catherine McGrath, Peter Andrews, Mark Lloyd

**Affiliations:** Frimley Park Hospital, Frimley, United Kingdom; Frimley Park Hospital, Frimley, United Kingdom; Frimley Park Hospital, Frimley, United Kingdom; Frimley Park Hospital, Frimley, United Kingdom; Frimley Park Hospital, Frimley, United Kingdom; Frimley Park Hospital, Frimley, United Kingdom

## Abstract

**Introduction:**

Although very rare, with an annual incidence of approximately 1 in 1 million, Takayasu arteritis (TAK) predominantly affects affects younger females. This makes issues related to child bearing particularly relevant to the management of the condition. The heterogenous nature of the condition, difficulty in assessing the degree of active vasculitis, and lack of guidelines specific for TAK in pregnancy make management challenging. We describe two cases managed in a district general hospital (DGH) with close interdepartmental and tertiary centre support which highlight these difficulties, though happily resulted in successful pregnancies.

**Case description:**

**Case 1**: TAK diagnosed in 2015 age 23. Presented with headaches and hypertension. MRA: focal and irregular stenosis of the abdominal aorta in the region of the renal arteries with focal stenosis of the left renal artery. Stenosis coeliac axis and superior mesenteric artery origins. Lupus anticoagulant positive (no thrombotic episodes). Initial management: prednisolone, mycophenolate 1 g bd, with rapid normalisation of CRP and weaning of prednisolone. Triple antihypertensive treatment. Joint management with tertiary centre. 2018: unsuccessful left renal angioplasty. Developed lower limb claudication. Despite normal PET-CT activity, concern that TA was active. MMF switched to MTX + tocilizumab. MDT decision to hold off reconstructive vascular surgery. 2019: unplanned ectopic pregnancy, managed with MTX. Following this MTX was switched to AZA. 2020: successful pregnancy - normal vaginal delivery with epidural on tocilizumab, AZA, prednisolone 5 mg daily, aspirin 75 mg daily. 2021: further successful pregnancy - elective LSCS at 39/40. Both deliveries at DGH.

**Case 2**: TAK diagnosed 2011 age 14 in Nepal. 2013: ascending aortic arch and hemi-arch surgery. Immigrated to the UK and seen by our team in 2023 at 23/40 gestation. Managed jointly with a tertiary centre. She remained on a long-term regimen of prednisolone 5 mg, metoprolol was increased from 12.5 mg daily to 25 mg daily and aspirin 75 mg daily was given. MRA showed mild dilatation from the distal aortic arch to the suprarenal aorta and diffuse narrowing of the both external iliac and superficial femoral arteries, left subclavian stenosis. Echocardiogram: moderate aortic incompetence. She developed gestational diabetes in the second trimester, growth scans showed no IUGR. Delivery was managed at a tertiary centre, with elective LSCS at term. There were no complications to mother or baby. Post delivery there was a mild increase in her moderate aortic incompetence.

**Discussion:**

TAK itself does not appear to affect fertility, nor does pregnancy affect TAK activity. However, pregnancy is high risk in TAK. A literature review of 505 pregnancies in 373 patients found life threatening complications in 5% and death in three cases. 35% developed hypertension, 16% births were premature, 15% had IUGR and there was a 12% foetal loss rate. Active TAK is associated with poor outcomes. In this sense both our patients were fortunate that, despite significant arterial abnormalities, their diseases were well controlled and they were able to proceed to term, delivering healthy babies. In Case 1 there was concern that the distal aortic stenosis could lead to reduced uterine blood flow; the successful outcome in both pregnancies suggests blood supply was adequate.

Case 1 required adjustment of potentially teratogenic medicines but illustrates that tocilizumab can be continued through pregnancy.

Pre-existing hypertension increases the risk of superimposed preeclampsia by 25%, necessitating the initiation of low-dose aspirin. Case 1 continued amlodipine 5 mg and doxazosin 4 mg twice daily during pregnancy. Amlodipine is a safe calcium channel blocker in pregnancy. Although limited data exist for doxazosin, one study indicated low foetal concentrations even when crossing the placenta at doses up to 8 mg, confirming the safety of our patient’s dose.

Case 2 involved a less complex treatment scenario, where continuous therapy with prednisolone was sufficient to maintain disease stability. However, post-pregnancy assessments indicated slight disease progression, necessitating ongoing vigilance.

MRA was utilised for monitoring disease activity, emphasising the importance of selecting imaging modalities that minimise radiation exposure to the foetus. The use of steroids can increase the risks associated with pregnancy, such as gestational diabetes, preeclampsia, preterm delivery, IUGR, and foetal loss. Fortunately, both patients were able to be managed on low dose prednisolone.

**Key learning points:**

• Pre-conception counselling is important in TAK, a disease often affecting females of child bearing age. This also guides choice of treatment and investigation. Both these patients were managed with very close collaboration between secondary and tertiary care centres. Unfortunately none of the centres had linked health records, highlighting the need for the continued development of nationally linked health IT systems.

• The lack of formal guidelines for TAK in pregnancy means that expert reviews are vital. We are fortunate that there are comprehensive UK guidelines on medication in pregnancy, as well as very supportive tertiary centres.

• The importance of a multidisciplinary team, including obstetricians and rheumatologists, is highlighted. Close monitoring and early referral to a tertiary centre are essential for managing complicated pregnancies.

• The relative rarity of TAK with pregnancy suggests the usefulness of a national registry for such cases.

